# Spatial clustering of fourteen tick species across districts of Zimbabwe

**DOI:** 10.1186/s12917-021-02792-2

**Published:** 2021-02-27

**Authors:** Munyaradzi Davis Shekede, Silvester Maravanyika Chikerema, Moregood Spargo, Isaiah Gwitira, Samuel Kusangaya, Aldridge Nyasha Mazhindu, Daud Nyosi Ndhlovu

**Affiliations:** 1 Department of Geography Geospatial Sciences and Earth Observation, Faculty of Science, University of Zimbabwe, MP167 Mount Pleasant, Harare, Zimbabwe; 2 Department of Clinical Veterinary Studies, Faculty of Veterinary Science, University of Zimbabwe, MP167 Mount Pleasant, Harare, Zimbabwe; 3 Department of Livestock and Veterinary Services, 18 Borrowdale Rd, Harare, Zimbabwe

**Keywords:** Tick species, Hotspots, Getis-Ord G_i_* statistic, Livestock disease transmission, Disease management

## Abstract

**Background:**

Ticks transmit several diseases that result in high morbidity and mortality in livestock. Tick-borne diseases are an economic burden that negatively affect livestock production, cost countries billions of dollars through vaccine procurement and other disease management efforts. Thus, understanding the spatial distribution of tick hotspots is critical for identifying potential areas of high tick-borne disease transmission and setting up priority areas for targeted tick disease management. In this study, optimised hotspot analysis was applied to detect hotspots and coldspots of 14 common tick species in Zimbabwe. Data on the spatial distribution of tick species were obtained from the Epidemiology Unit of the Division of Veterinary Field Services of Zimbabwe.

**Results:**

A total of 55,133 ticks were collected with *Rhipicephalus decoloratus* being the most common species (28.7%), followed by *Amblyomma hebraeum* (20.6%), and *Rhipicephalus sanguineus* sensu *lato* (0.06%) being the least common species. Results also showed that tick hotspots are species-specific with particular tick species occupying defined localities in the country. For instance, *Amblyomma variegatum*, *Rhipicephalus appendiculatus*, *Rhipicephalus decoloratus, Rhipicephalus compostus, Rhipicephalus microplus*, *Rhipicephalus pravus*, and *Rhipicephalus simus* were concentrated in the north and north eastern districts of the country. In contrast, *Amblyomma hebraeum*, *Hyalomma rufipes, Hyalomma trancatum* and *Rhipicephalus evertsi evertsi* were prevalent in the southern districts of Zimbabwe.

**Conclusion:**

The occurrence of broadly similar hotspots of several tick species in different districts suggests presence of spatial overlaps in the niche of the tick species. As ticks are vectors of several tick-borne diseases, there is high likelihood of multiple disease transmission in the same geographic region. This study is the first in Zimbabwe to demonstrate unique spatial patterns in the distribution of several tick species across the country. The results of this study provide an important opportunity for the development of spatially-targeted tick-borne disease management strategies.

## Background

Ticks host pathogens that cause several livestock diseases of veterinary and economic concern [1]. The pathogens include bacteria, helminths, protozoans and viruses. These pathogens cause a variety of diseases that negatively affect livestock production and human health [2]. For instance, tick borne diseases affect 80% of the world cattle population [3], cost countries between 13.9–18.7 billion annually through vaccine procurement and deaths (35). The high cattle morbidity and mortality result from common tick-borne diseases such as anaplasmoses, babesioses, cowdriosis and theilerioses [4]. In regions where tick-borne diseases are common, mortality rates in the range of 20–95% have been recorded. For example, in sub-Saharan Africa theileriosis (East Coast Fever) alone accounts for ~ 1 million cattle deaths annually resulting in approximately USD300 million in economic losses [5]. The high mortality rates are associated with low dipping frequency prevalent in communal areas where farmers depend on government assistance for chemicals [6]. In addition, ticks reduce growth rates and milk production as well as induce fertility problems among livestock [7]. Although ticks mostly affect livestock, they also threaten human health in instances where people consume meat from animals that succumb to diseases spread by ticks [1, 8]. Thus, understanding the spatial distribution of tick hotspots, i.e., geographic regions with high tick prevalence, is important for identifying potential tick-borne disease transmission areas. Information on tick hotspots is critical for guiding livestock disease control and management strategies such as optimal location of dip tanks. Moreover, mapping tick hotspots is critical for optimal resource allocation through targeting preventive and control strategies to areas with greatest need. This is particularly important in resource limited countries such as Zimbabwe where dipping chemicals are usually inadequate to cover the whole country.

Several studies have been undertaken on various aspects of ticks and tick-borne diseases in Zimbabwe. These studies range from those that characterised life cycle of tick parasites [9] to those that modelled suitable habitats of tick vectors [1, 10]. Related studies focused on tick-borne disease control [11–14], experimental vaccine trials in cattle [1] including tick infestations among livestock [6]. Other studies concentrated on communal farmers’ perceptions of cattle diseases [7, 15] and disease epidemiology [6, 16]. While these studies provided invaluable insights into tick-borne disease transmission, they lack location-specific information on the spatial distribution and prevalence of each tick species. This is despite the fact that location-specific information on different tick species forms the basis for effective management of tick-borne diseases. In fact, spatial patterns of tick prevalence have not been fully explored in Zimbabwe [17]. The limited focus on spatial patterns of tick prevalence may partly explain why, to date, there is no consistent strategy to effectively prevent or control tick-borne diseases in the country [18]. In this regard, modelling spatial patterns of tick prevalence through hotspot analysis is critical for understanding livestock disease ecology as well as providing knowledge on parasite transmission dynamics that is important for effective and sustainable management of tick-borne diseases.

Modelling of spatial patterns of tick hotspots has improved greatly over the years due to the ready availability of spatially referenced data on tick species prevalence across Zimbabwe. The increased availability of tick species data coupled with advances in geospatial tools and spatial statistics allow for the determination of location specific patterns including their evolution over time. In a recent study by [19], changes in the spatial distribution of lumpy skin disease hotspots were modelled using georeferenced data in Zimbabwe. Spatial statistics were applied to detect transmission hotspots of human anthrax in Georgia in a study by [20]. Furthermore, [17] used a retrospective space–time scan statistics to detect temporal and spatial clusters of dermatophilosis, a tick-borne disease. Put together, these studies provide important insights into the importance of applying geospatial tools and spatial analytical approaches in improving tick and tick-borne disease hotspot detection. In this study, therefore, geospatial techniques and spatial statistics were applied to explore the spatial distribution and prevalence of 14 georeferenced tick species. The intention was to test whether the occurrence of the tick species exhibited any systematic spatial pattern as opposed to being randomly distributed in Zimbabwe.

## Results

### Spatial distribution of tick hotspots in Zimbabwe

A total of 55,133 ticks were observed across the sampled districts with *Rhipicephalus decoloratos* (28.85%) being the dominant species, followed by *A. hebraeum* (20.6%), *R. appendiculatus* (14.8%), *H.rufipes* (8.9%), *R. microplus* (7.2%), *R. evertsi evertsi* (5.9%), *A.variegatum* (3.8%), *H. truncatum* (2.7%), *R. simus* (2.3%), *R. zambeziensis* (2.2%), *R. compostus* (2.2%), *H. leachi* (0.4%), *R. pravus* (0.09%) and *R. sanguineus* sensu *lato* (0.06%).

**Fig. 1:**
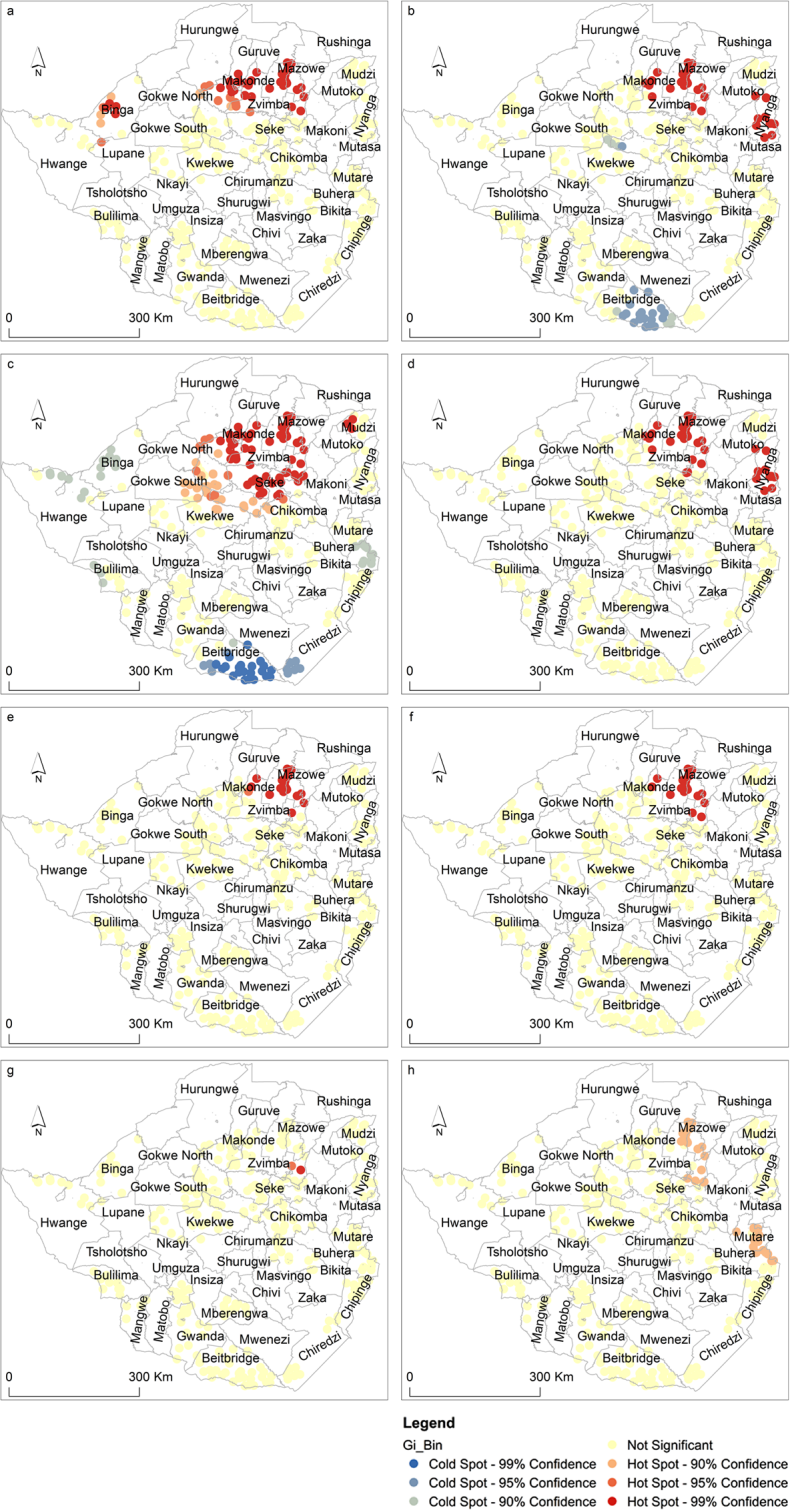
Spatial distribution of **a** *Amblyomma variegatum,* **b** *Rhipicephalus appendiculatus*, **c** *Rhipicephalus decoloratus*, **d** *Rhipicephalus compostus,* **e** *Rhipicephalus microplus*, **f** *Rhipicephalus simus*, **g** *Rhipicephalus zambeziensis* and **h**, *Rhipicephalus pravus* hotspots and coldspots across districts of Zimbabwe (Map designed and produced by the authors). The figure illustrates the spatial distribution of hotspots and cold spots across districts of Zimbabwe

**Fig. 2:**
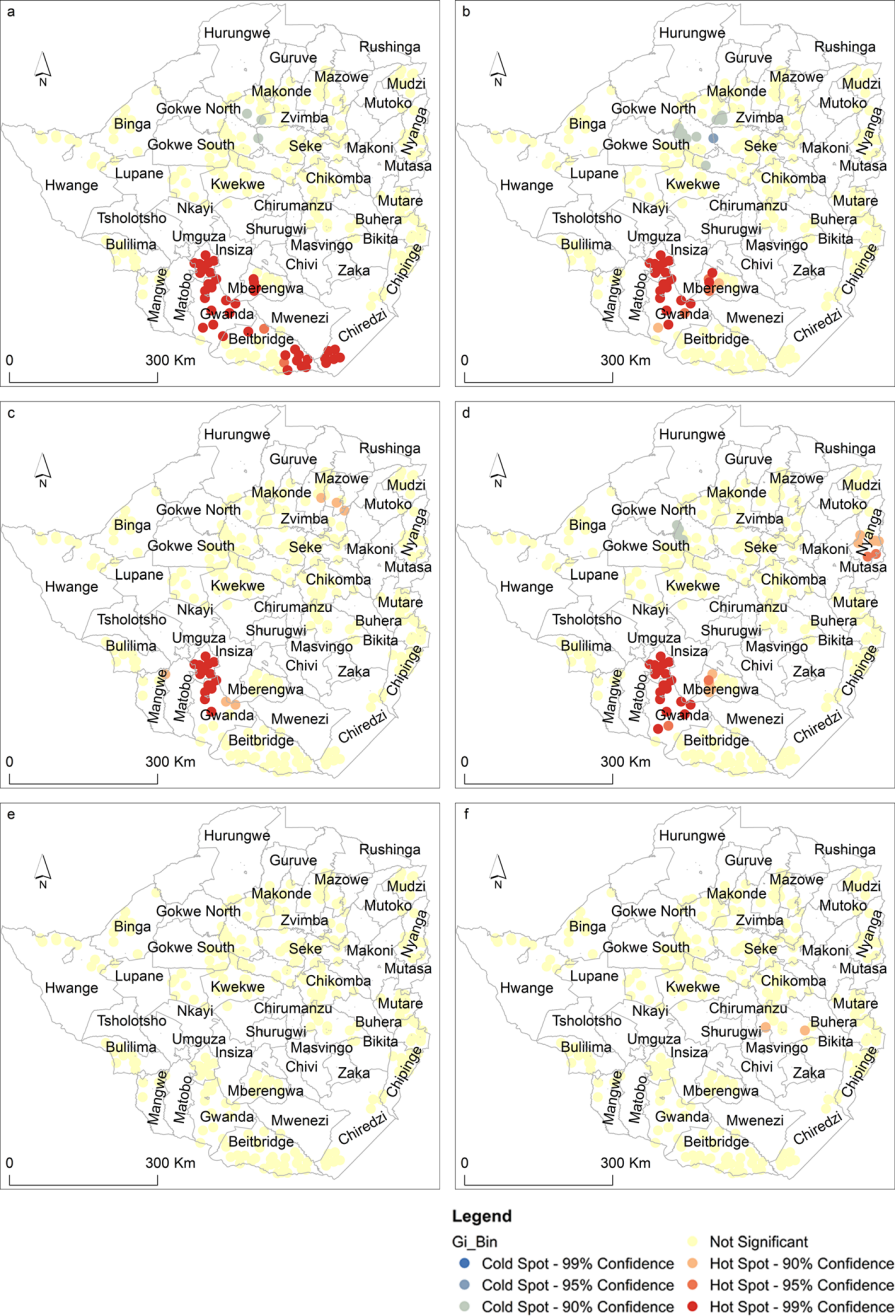
Spatial distribution of **a** *Amblyomma hebraeum*, **b** *Hyalomma rufipes* **c**, *Hyalomma trancatum*, **d** *Rhipicephalus evertsi evertsi* **e** *Rhipicephalus decoloratus* and **f** *Rhipicephalus sanguineus* sensu *lato* hotspots and coldspots in Zimbabwe (Map designed and produced by the authors). The figure illustrates the spatial distribution of hotspots and cold spots across districts of Zimbabwe

The spatial distribution of tick hotspots across districts of Zimbabwe is illustrated in Figs. 1 and 2. The results indicate that particular tick species tend to cluster in specific localities in the country. For instance, the north and north eastern districts of the country are characterised by spatial clustering of *A. variegatum, R. appendiculatus*, *R. decoloratus*, *R. compostus, R. microplus*, *R. simus*, *R. zambeziensis* and *R. pravus*. Specifically, the eight tick species were dominant in Bindura, Goromonzi, Guruve, Mazowe, Mudzi, Nyanga, Shamva and Zvimba districts (Fig. 1). The spatial distribution of *A. variegatum* (a), *R. appendiculatus* (b), *R.decoloratus* (c), and *R. compostus* (d) was more extensive than other tick species (Fig. 1).

In contrast, Fig. 2 shows that hotspots of *A. hebraeum* (a), *H. rufipes* (b), *H. trancatum* (c) and *R. evertsi evertsi* (d) tick species were prevalent in the southern districts of Zimbabwe. These tick species were common in Beitbridge, Chiredzi, Gwanda and Umzingwane districts.

Furthermore, *R. appendiculatus* (Fig. 1b) and *R. decoloratus* (Fig. 1c) had identifiable coldspots in the southern regions of the country. Compared to other tick species that formed identifiable hotspots or coldspots, *H. leachi* (Fig. 2e) and *R. sanguineus* sensu *lato* (Fig. 2f) exhibited a random pattern.

## Discussion

The study aimed at testing whether 14 tick species located in Zimbabwe exhibited significant clustering based on spatial statistics. Results of this study showed that most tick species exhibit hotspots and coldspots in specific districts of the country. For instance, seven tick species i.e., (Fig. 1) (a) *A. variegatum*, (b) *R. appendiculatus*, (c) *R*. *decoloratus*, (d) *R. compostus*, (e) *R. microplus*, (f) R. *simus* and (g) R.*zambeziensis*) had hotspots predominantly located in the north and north eastern districts of the country while four (Fig. 2)(a) Amblyomma *hebraeum*, (b) *H. rufipes* (c), *H. trancatum* and (d) *R. evertsi evertsi*) had significant hotspots in the southern districts. This study is the first to quantitatively detect hotspots of several tick species in Zimbabwe within a spatially-explicit analytical framework.

The spatial coincidence in the occurrence of tick species hotspots e.g., *A. variegatum*, *R appendiculatus* and *R. decoloratus* in the northern parts of the country, suggests spatial overlaps in the niche of these species. Spatial overlaps were also noted in the southern parts of the country associated with ticks such as *A. hebraeum*, H. rufipes and H. truncatum. The presence of spatial overlaps in the geographic distribution of these tick species suggests the possibility of co-infection of livestock by several tick-borne diseases. This calls for an integrated approach in the control of multiple tick-borne diseases in areas characterised by multiple tick species. In addition, the spatial overlaps in hotspots and, through inference, habitats of tick vectors imply the need for further research aimed at understanding the common drivers of these ticks [21]. Results of this study therefore, provide valuable insights into the need to develop novel, spatially explicit control and prevention strategies for tick-borne diseases in similar environments. For instance, this study showed that the southern parts of the country are not hotspot areas for *R. appendiculatus* which is an important vector of theileriosis in Zimbabwe. The implication therefore is that tick control measures in this particular region, should be targeted at *A. hebraeum* and *R. decoloratus* whose life cycles on the host are longer than that of *A appendiculatus* [1]. This study corroborates previous studies on the distribution of particular tick species such as *A. hebraeum*, *R. appendiculatus*, *R. microplus* and *R. decoloratus*. A study by [21] reported that the distribution of *A. variegatum* was mainly limited to the north western parts of Zimbabwe while *A. hebraeum* was mainly found in the southern parts of the country.

In this study, results show that the occurrence of ticks such as *H. leachi* and *R. sanguineus* sensu *lato* exhibit a random pattern. In other words, the specific tick species are randomly distributed in space and thus do not form any significant clusters. Such a pattern in tick distribution pose challenges in the implementation of control and prevention strategies as the vectors do not have a specified location which can be targeted. The results imply the need for constant monitoring of such ticks to detect any potential outbreaks in diseases associated with them. The Government of Zimbabwe’s Animal Health (Cattle cleansing) Regulations Statutory Instrument (SI) 250 of (1993) specifies tick species such as *R. appendiculatus*, *A. hebraeum*, *A. variegatum*, *R. decoloratus* and *R. microplus* as pests hence dipping or any tick control strategy should target such species. The presence of hotspots of particular tick species in certain districts might require these areas to be declared as endemic for purposes of enhanced tick and tick-borne disease control.

**Fig. 3:**
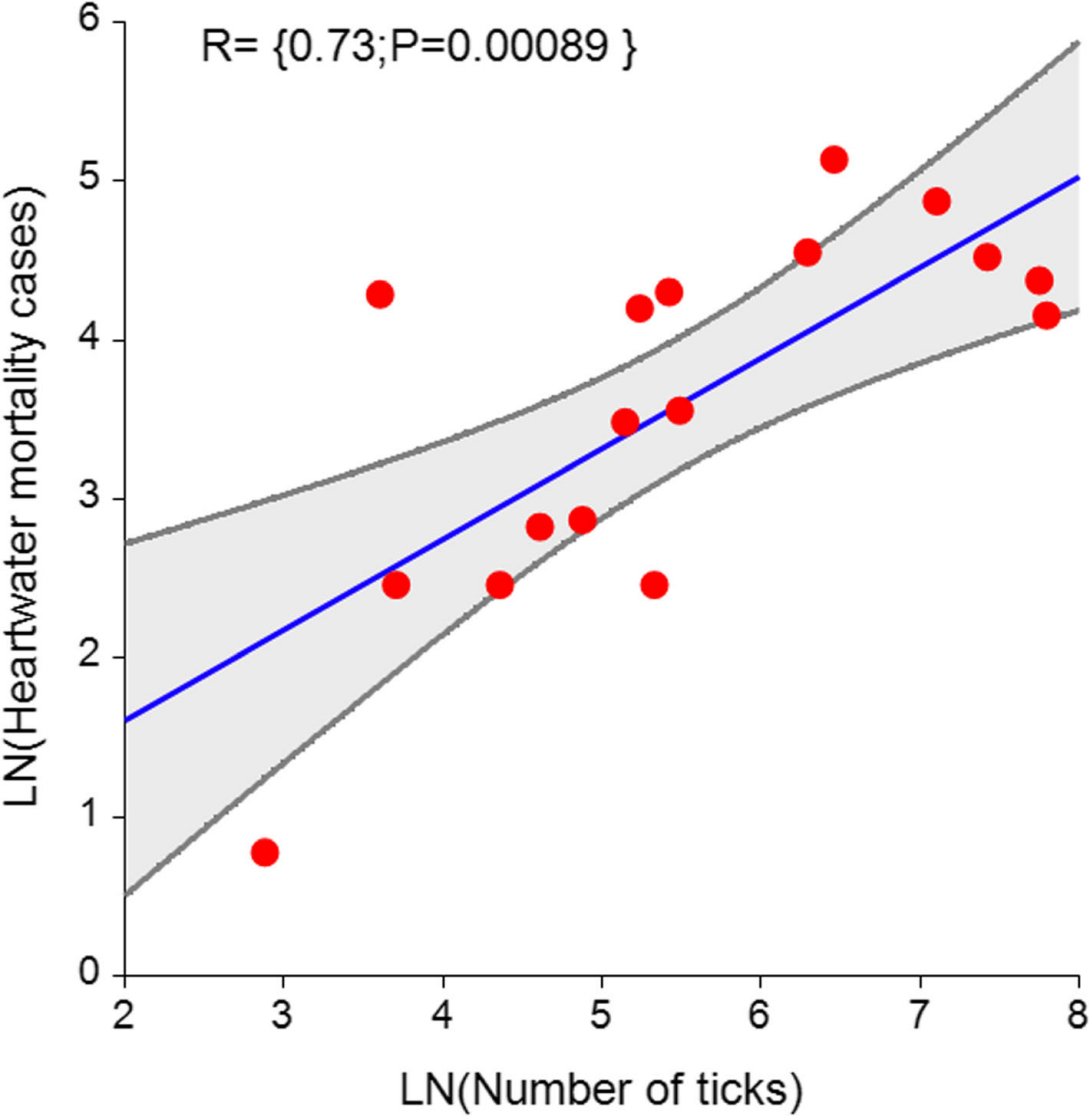
The significant positive relationship between Amblyomma hebraeum abundance and cattle mortality from heart water disease (Figure designed and produced by the authors). The figure illustrates relationship between Amblyomma hebraeum abundance and cattle mortality from heart water disease

The spatial clustering of ticks in specific districts corroborate and expand previous studies on the spatial distribution of ticks and tick-borne diseases in Zimbabwe. In a previous study, [17] modelled spatio-temporal patterns of bovine dermatophilosis (a disease associated with *A variegatum)* over a 19 year period in Zimbabwe and observed a directional spread of the disease from the identified clusters over time. Similarly, [19] showed that there was a shift in the distribution of ixodid ticks in Zimbabwe. While these studies and other related studies have generated useful information on areas to target for disease management, results of this study emphasise the need for increasing efforts towards understanding tick distribution and its related local epidemiological significance. In fact, this study observed a strong significant positive correlation between *A. hebraeum* abundance and cattle mortality resulting from heartwater disease (r = 0.73, *p* = 0.000) thereby further emphasising the need for tick hotspot detection (Fig. 3). Thus, focusing on the source of tick-borne diseases is likely to be more effective in preventing the diseases than trying to cure the disease.

Although mechanisms explaining hotspots/coldspots of specific tick vector species were beyond the scope of this study, further investigation is required to understand the drivers of detected patterns. However, the prevalence of tick species in a geographic area is driven by factors such as dipping frequency, acaricide resistance, unrestricted movement of cattle, availability of alternative hosts, rainfall patterns and landuse changes. For example, reduced dipping frequency is known to promote endemism of tick-borne diseases [22]. In Zimbabwe, dipping frequency declined significantly over the years due to a shift in responsibility of tick control from government to livestock owners in line with economic structural adjustment programs [7] coupled with foreign currency shortages for procurement of dipping chemicals. The reduced dipping frequency has been attributed to the death of approximately 50,000 cattle as a result of theileriosis during the 2017/2018 rainfall season. Furthermore, acaricide resistance has been identified as a key threat to control of tick vectors in Zimbabwe especially in geographic settings where acaricide rotation practices are absent [7]. Land use change and shifting rainfall patterns across different agro-ecological regions are also likely to result in spatial heterogeneity in tick prevalence at various spatial scales [21]. The approach adopted in this study and the associated results could be key in influencing the need for a change in the traditional approach of tick-borne disease management where similar strategies of tick control are implemented throughout the country. Based on the results of this study, dipping frequency could be guided by abundance and distribution of different tick species. Further, this information can be used to strengthen existing legislation on tick control.

A potential weakness of this study is in its relatively short duration (five months) and insensitivity to the life stage of ticks in the analysis of hotspots. It is possible that increasing sampling time could correspondingly increase the number of tick species encountered. For instance, a previous study [23] reported encountering 15 tick species during a one and half year survey. Further, increasing study duration could be important in exploring population dynamics and spatial distribution of tick species across the country. Given the seasonality of rainfall in the country, future studies could test whether the spatial distribution of ticks is related to season in addition to determining whether hotspots are life-stage dependent. Nonetheless, results of this study are useful in targeted management of tick-vectors which is relevant in resource constrained countries.

## Conclusion

This study tested the extent to which fourteen tick species cluster in space across districts of Zimbabwe. Results indicate the co-occurrence of hotspots for several tick species suggesting possible spatial overlaps in their niche in addition to possible co-infection of livestock by multiple diseases. This study is one of the first in Zimbabwe to demonstrate unique capabilities of spatial statistics in understanding distribution of several tick species in specific parts of the country. Results of this study provide opportunities for the development of tick-borne disease management and control strategies tailored for specific areas.

## Methods

### Study area

The study was carried out across districts of Zimbabwe; a semi-arid country located between 15°30″ and 22°30″S of latitude 25°00″ and 33°10″E longitude. Elevation is highest in the eastern parts of the country (> 2500 m above mean sea level) and lowest in the southern and northern parts of the country where it reaches less than 300 m a.s.l. The climate is characterised by three main seasons i.e., 1) cool dry season from May to August, 2) hot and dry season between August and October and a hot and wet season stretching from November to April [24]. On a temporal scale, temperatures in the country range from an average low of ~ 15 °C July to around 24 °C in November. There is a discernible spatial dimension in temperature moderated by altitude where the eastern highlands experience the lowest mean annual temperature of 18 °C while the northern and southern low lying areas experience the highest temperatures of around 23 °C. Rainfall ranges from an annual average of less than 400 mm in the western and southern parts of the country to > 1500 mm in the Eastern highlands [25].

### Data sources

#### Tick species data

**Fig. 4:**
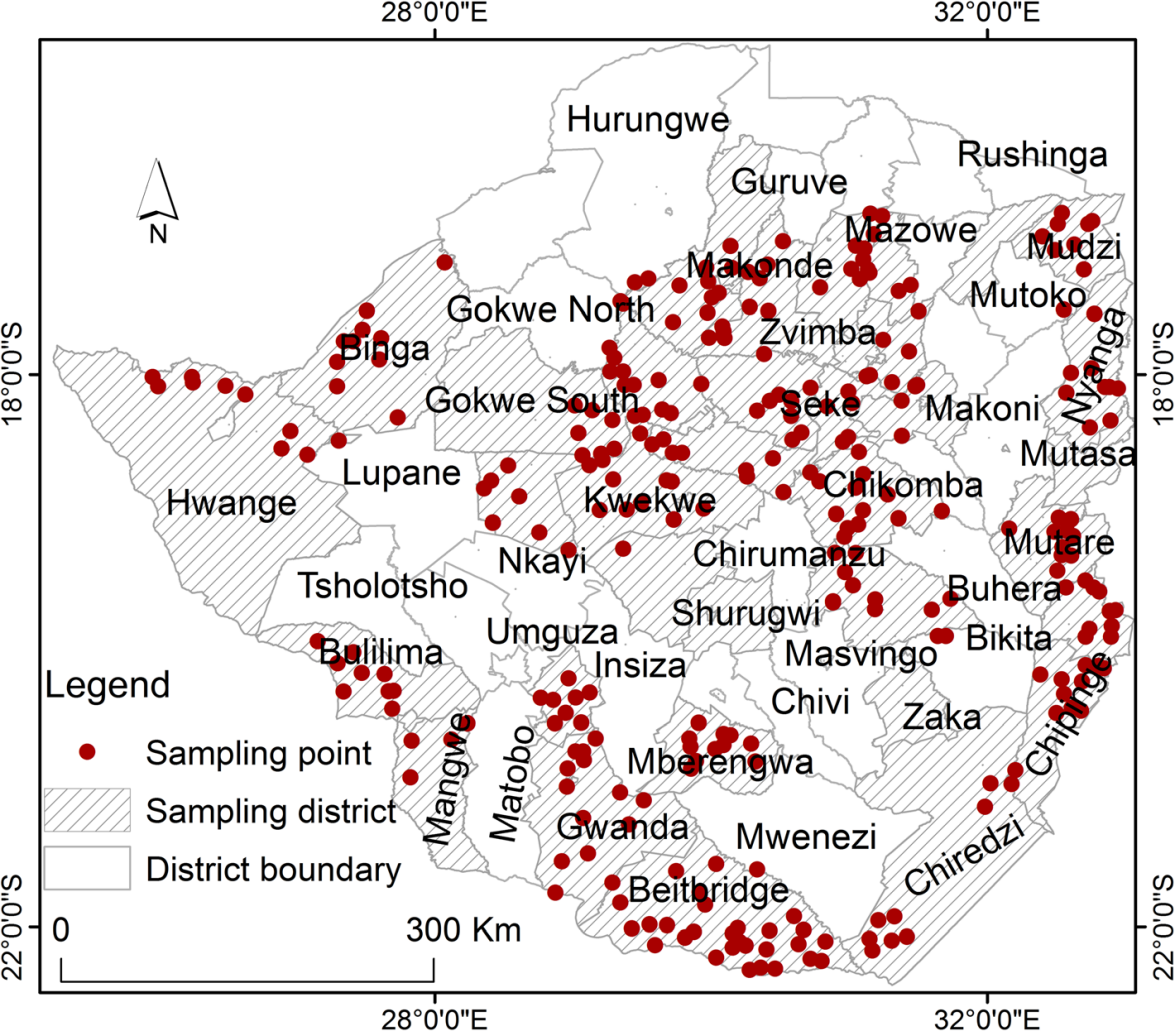
Location of sampling points within the 27 districts (Map designed and produced by the authors). The figure illustrates the sampling points within the 27 sampled districts of Zimbabwe

In this study, spatially referenced data of fourteen [14] tick species were obtained from the Epidemiology Unit of the Division of Veterinary Field Services. The data were from a national survey carried out between December 2011 and April 2012 at 303 dip tanks in the farming (non-urban) districts of country, with at least 10 dip tanks being selected from each district (Fig. 4). Stratified random sampling was applied in the selection of sample districts with agro-ecological regions used as the stratum. Thereafter, a minimum of 10 dip tanks were randomly selected within each of the selected districts. Half of the farming districts [26] with 303 dip tanks were finally selected for sampling taking into account accessibility as well as resource availability (Fig. 4). During the survey, trained veterinary personnel collected the ticks from one side of the cattle host using forceps. Ticks from the ear canal were collected by scrapping the canal using a curratte, and thereafter the ticks were placed in 70% ethanol before being taken to the Central Veterinary Laboratory, Harare. Morphological identification to species level was carried out by trained veterinarians using standard taxonomic keys [26, 27] with the aid of a standard stereomicroscope. Specifically, the keys provide a description of morphological attributes such as body shape, size and texture that are used for tick species identification through visual matching of specimens to drawings or pictures using a dissecting microscope among other techniques of species identification.

### Data analysis

#### Hotspot analysis

The Getis G_i_* statistic [28, 29] was used to test whether the distribution of each of the fourteen tick species significantly cluster in space across Zimbabwe.

The Getis Gi* statistic is calculated using the following formula:

$$ {\mathrm{G}}_{\mathrm{i}}^{\ast }(d)=\frac{\sum j\  Wij\ (d) xj- Wi\ast \overline{x}}{s^{\ast \left\{\left[\left(n{S}^{\ast } li\right)-{W_i}^{\ast 2}\right]/\left(n-1\right)\right\}}1/2} $$

where.

*Wij (d)* is a spatial weight vector with values for all cells ‘*j*’ within distance d of target cell *i*, *W*_i_^*^ is the sum of weights, S*li is the sum of squared weights and s* is the standard deviation of the data in the cells.

The Getis-Ord G_i_* is a spatial statistical method available with optimized hotspot analysis function in ArcGIS 10.2 [30]. Three confidence interval (CI) levels (90, 95, and 99%) were used, and higher confidence levels imply intense aggregation of hotspots or coldspots in the occurrence of ticks [31]. The G_i_* statistic was implemented through comparing the abundance of a particular tick species at a particular location in relation to the abundance of the same species in the surrounding points. Specifically, the G_i_* statistic was implemented in a GIS environment by first computing the local sum of the observed number of ticks of a given species at a given point and its neighbouring points before proportionally comparing these to the sum of all ticks of the same species observed in the study area [32]. Next the difference between local sum and the expected local sum under the null hypothesis of complete spatial randomness was calculated to yield a statistically significant Z-score. When the difference between the two is too large to be a result of chance then the area is designated as a hotspot [28, 29, 33]. The result of Getis-Ord G_i_* analysis is a Z-score where if the Z score is positive and significant, then an area is characterised by a relatively high occurrence of ticks (hotspot). In contrast, if the Z-score is negative and significant, it indicates a coldspot (Ord and Getis, 1995, 2001). Thus, clustering refers to higher number of observed tick species than would be expected by chance in space (hotspots) or fewer than expected tick cases (coldspots) [34]. Areas with Z scores > 1.96 were considered as significant at 99% confidence level (*p* < 0.01), and classified as hotspots while areas characterised by Z-scores of <− 1.96 indicated clustering of low tick occurrences hence were classified as coldspots [31]. The same procedure was performed on each of the fourteen species to yield fourteen hotspot maps.

The Getis-Ord G_i_* was selected among several spatial clustering techniques used to detect hotspots, due to its simplicity in detecting hotspots. Despite the drawback in the method such as the problem of multiple testing and decision criteria (as to whether an area is a hotspot or not), the technique has been successfully used to detect hotspots in different geographic areas [35–37].

## Data Availability

The datasets used and/or analysed during the current study are available from the corresponding author on reasonable request.
